# Clinical and Radiographic Factors Associated With Rotator Cuff Tears: A Case-Control Study

**DOI:** 10.7759/cureus.106706

**Published:** 2026-04-09

**Authors:** Zied Masmoudi, Sofiane Masmoudi, Mohamed Ali Khlif, Khaled Zitouna, Hend Riahi, Maher Barsaoui

**Affiliations:** 1 Orthopaedics, Faculty of Medicine of Tunis, Tunis El Manar University, Tunis, TUN; 2 Radiology, Faculty of Medicine of Tunis, Tunis El Manar University, Tunis, TUN

**Keywords:** acromial index, body mass index, critical shoulder angle, occupational exposure, risk factors, rotator cuff injuries

## Abstract

Background

Rotator cuff tears (RCTs) are considered multifactorial, arising from both intrinsic and extrinsic mechanisms. The objective of this study was to evaluate the association between 12 clinical and radiographic factors, including smoking, alcohol consumption, occupational exposure, dominant side involvement, prior shoulder trauma, dyslipidemia, diabetes, thyroid dysfunction, obesity, arterial hypertension, critical shoulder angle (CSA), and acromial index (AI), with the occurrence of RCTs and to identify which factors independently predict their development.

Methodology

This retrospective, case-control study included patients with RCTs confirmed intraoperatively and age and sex matched controls with ultrasound-confirmed intact rotator cuffs. Clinical variables were recorded, and radiographic parameters (CSA and AI) were measured on standardized anteroposterior shoulder radiographs. Multivariable logistic regression analysis was performed to identify independent predictors, and receiver operating characteristic (ROC) curve analysis was used to assess the diagnostic performance of radiographic parameters.

Results

In total, 80 participants were included: 40 cases (50%) and 40 matched controls (50%). RCTs were significantly associated with higher body mass index (BMI) (p = 0.003), dominant side involvement (p = 0.007), high-risk occupation (p = 0.001), higher AI (p = 0.003), and higher CSA (p = 0.005). Dyslipidemia, diabetes, dysthyroidism, arterial hypertension, alcohol consumption, smoking, and a history of shoulder trauma were not significantly associated with RCTs. ROC curve analysis showed CSA had superior predictive performance compared with AI. Multivariable logistic regression showed that CSA, BMI, dominant side involvement, and a high-risk occupation remained significantly and independently associated with the occurrence of an RCT. The AI was not an independent predictor.

Conclusions

RCTs are associated with both anatomical and clinical factors, particularly CSA, BMI, occupational exposure, and dominant side involvement. Among radiographic parameters, the CSA demonstrated the strongest predictive value. These findings may contribute to improved risk stratification and preventive strategies, particularly regarding weight management and workplace ergonomics, although prospective studies are required to confirm these associations.

## Introduction

Chronic shoulder pain is a leading reason for orthopedic consultation and ranks as the third most frequent musculoskeletal complaint worldwide [1]. Among its primary etiologies, rotator cuff tears (RCTs) are considered the leading cause [2].

RCT development is widely considered multifactorial. Two principal mechanisms have traditionally been described. The intrinsic theory implicates degenerative tendon changes related to aging and progressive hypovascularization [3], whereas the extrinsic theory emphasizes the role of acromial morphology in the development of subacromial impingement [4].

Recent literature has further expanded this framework by identifying specific scapular morphological markers. These include increased lateral acromial extension, measured by the acromial index (AI), and increased glenoid inclination, quantified by the critical shoulder angle (CSA) [5,6]. Furthermore, a growing body of evidence suggests that RCT occurrence is influenced by a complex interplay of socioeconomic factors such as occupational exposure, limb dominance, and history of trauma, alongside clinical and metabolic conditions, including dyslipidemia, diabetes, thyroid dysfunction, obesity, and hypertension [7-12].

The objective of this study was to evaluate the association between 12 clinical and radiographic factors, including smoking, alcohol consumption, occupational exposure, dominant side involvement, prior shoulder trauma, dyslipidemia, diabetes, thyroid dysfunction, obesity, arterial hypertension, CSA, and AI, with the occurrence of RCTs and to identify which factors independently predict their development.

## Materials and methods

Study design

This was a retrospective, case-control, single-center study conducted on two patient groups: a case group with confirmed RCT, and a control group matched by age and sex, with an intact rotator cuff. Patients were consecutively identified from institutional records over the study period (January 2010 to June 2021) and screened according to predefined inclusion and exclusion criteria. Controls were matched to cases by age and sex, with age matching performed within a ±2-year range.

Inclusion and exclusion criteria

The inclusion and exclusion criteria are summarized in Table 1. In summary, the case group included adult patients (>18 years) with RCTs confirmed intraoperatively, while the control group consisted of patients with ultrasound-confirmed intact rotator cuffs. Patients with prior shoulder surgery, fractures, dislocations, or osteoarthritis were excluded.

**Table 1:** Inclusion and exclusion criteria of case and control groups.

Group	Inclusion criteria	Exclusion criteria
Case group	Older than 18 years old	Cervicobrachial neuralgia
	Have a standard anteroposterior radiograph	Prior shoulder surgery
	Rotator cuff tear confirmed intraoperatively	Isolated long head of biceps tears
		History of shoulder fracture/dislocation/osteoarthritis
Control group	Have a standard anteroposterior radiograph	Prior shoulder surgery
	Ultrasound-confirmed rotator cuff integrity	History of shoulder fracture/dislocation

Collected data

The recorded variables were selected based on factors previously reported in the literature to be associated with RCTs. These included demographic characteristics such as age and sex; clinical factors including diabetes mellitus, arterial hypertension, thyroid dysfunction, dyslipidemia, and obesity; lifestyle factors including smoking and alcohol consumption; and exposure-related variables including history of shoulder trauma, dominant side involvement, and occupational exposure.

Occupations were considered high risk if they involved repetitive overhead activities, sustained use of the upper limbs above shoulder level, or repetitive mechanical loading of the shoulder likely to induce microtrauma. Obesity was defined using body mass index (BMI), calculated as weight in kilograms divided by height in meters squared, with a BMI of 30 kg/m² or greater considered indicative of obesity. Radiographic parameters included the CSA and AI, both measured on standard anteroposterior shoulder radiographs.

All data were retrospectively extracted from electronic medical records and imaging archives using standardized data collection forms.

Radiographic evaluation

All imaging studies were performed as part of routine clinical care and retrospectively retrieved from institutional archives. Inclusion required standard anteroposterior shoulder radiographs for both groups and ultrasound-confirmed rotator cuff integrity in the control group. In the case group, RCTs were confirmed intraoperatively, serving as the reference standard. Patients undergoing surgery systematically received preoperative MRI or CT arthrography to confirm and characterize the lesion.

All imaging studies were interpreted by the same experienced radiologist, and in the control group, ultrasound was considered sufficient due to its high sensitivity when performed by an experienced operator [13].

**Figure 1:**
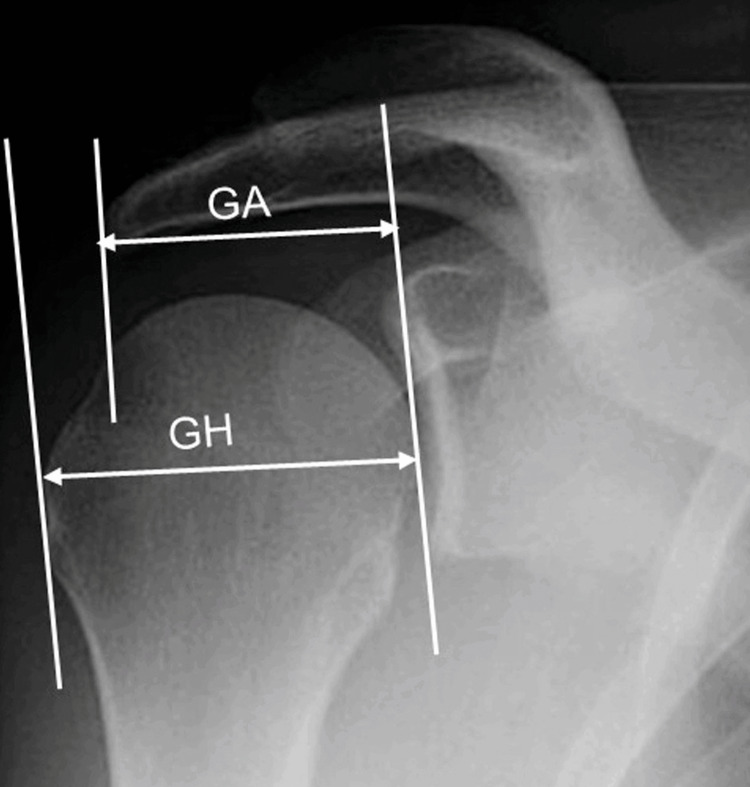
Acromial index measurement. The acromial index was determined by calculating the GA/GH ratio. GA represents the distance from the glenoid plane to the lateral edge of the acromion, and GH represents the distance from the glenoid plane to the lateral aspect of the humeral head.

**Figure 2:**
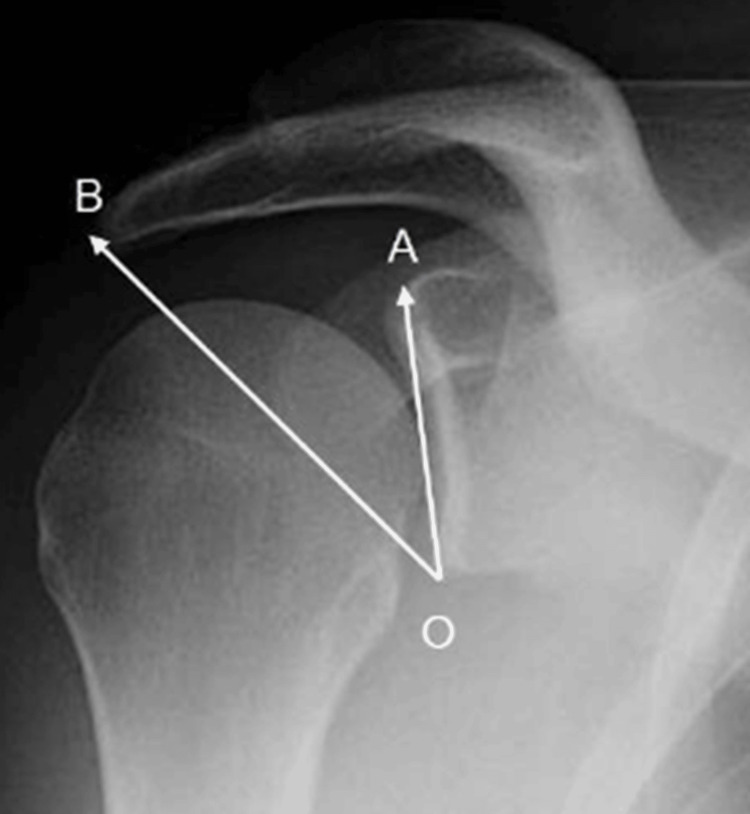
Critical shoulder angle measurement. The critical shoulder angle is the angle formed between the glenoid axis (OA) and the line connecting the inferior glenoid margin to the inferolateral border of the acromion (OB).

Radiographic parameters were measured manually by a single observer, using a goniometer on anteroposterior shoulder radiographs. The measured parameters included the AI, defined as the ratio between the distance from the glenoid plane to the lateral edge of the acromion and the distance from the glenoid plane to the lateral aspect of the humeral head (Figure 1) [5], and the CSA, defined as the angle between the superior and inferior bone margin of the glenoid and the most inferolateral border of the acromion (Figure 2) [14].

Statistical analysis

Statistical analysis was performed using SPSS version 26 (IBM Corp., Armonk, NY, USA). Qualitative variables were expressed as frequencies and percentages. Normality of continuous variables was assessed using the Shapiro-Wilk and Kolmogorov-Smirnov tests. Normally distributed variables were expressed as mean ± standard deviation (SD), while non-normally distributed ones were expressed as median and interquartile range (IQR).

Comparisons between groups were performed using Student’s t-test for normally distributed continuous variables and the Mann-Whitney-Wilcoxon test for non-parametric variables. Qualitative variables were compared using Pearson’s chi-square test or Fisher’s exact test when appropriate. Statistical significance was set at p-values <0.05. Receiver operating characteristic (ROC) curve analysis was used to evaluate the diagnostic performance of AI and CSA.

Variables with p-values <0.05 in univariate analysis were entered into the multivariable logistic regression model. Multicollinearity was assessed before model construction, and no significant collinearity was identified.

**Figure 3:**
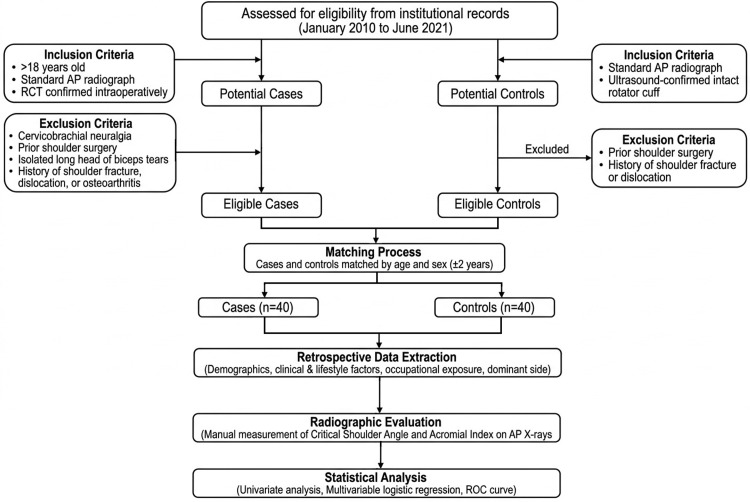
Flowchart of the study. AP = anteroposterior; RCT = rotator cuff tear; ROC = receiver operating characteristic

No significant missing data were identified for the variables included in the analysis. No prior sample size calculation was performed due to the retrospective nature of the study. The study flow chart is illustrated in Figure 3.

Ethical considerations

This study was conducted in accordance with the ethical principles of the Declaration of Helsinki. According to the local policy of the Faculty of Medicine of Tunis, formal approval from the institutional review board was not required for retrospective studies involving anonymized data. The requirement for written informed consent was waived due to the retrospective analysis of de-identified data, routinely collected in clinical practice.

## Results

In total, 80 patients were included, with 40 (50%) cases in each group. The mean age was 56.85 ± 9.82 years in the case group and 57.08 ± 9.38 years in the control group. The total population included 52 (65%) women and 28 (35%) men, evenly divided between the two groups. Comorbidities included diabetes in 27 (34%) patients, hypertension in 30 (38%), dyslipidemia in 30 (38%), and hypothyroidism in four (5%). Overall, 49 (61%) patients held a high-risk occupation. The most represented high-risk occupations in the case group were agricultural workers (six patients, 15%), construction workers (three patients, 7.5%), housekeepers (three patients, 7.5%), and truck drivers (three patients, 7.5%).

A history of shoulder trauma was reported by 27 (34%) patients: 16 (40%) in the case group and 11 (28%) in the control group. Pain involved the dominant side in 62 (78%) patients: 36 (90%) in the case group compared with 26 (65%) in the control group. In total, 16 (20%) patients were smokers, and seven (9%) consumed alcohol regularly.

The median BMI was 28.2 kg/m² (IQR = 25.6-30.8) in the case group and 25.8 kg/m² (IQR = 23.9-27.7) in the control group. The median CSA measured 38.3° (IQR = 36.2-40.1°) in cases and 34.3° (IQR = 30.6-38.2°) in controls. The median AI was 0.72 (IQR = 0.67-0.75) in the case group compared with 0.66 (IQR = 0.60-0.73) in the control group.

Pearson’s chi-square test revealed no significant association between RCT and diabetes (p = 0.24), hypertension (p = 0.64), dyslipidemia (p = 0.36), thyroid disease (p = 0.61), history of shoulder trauma (p = 0.24), smoking (p = 0.58), or alcohol (p = 0.99). Significant associations were found between RCT and dominant side involvement (p = 0.007), higher BMI (p = 0.003), high‑risk occupation (p = 0.001), higher CSA (p = 0.005), and higher AI (p = 0.003) (Table 2).

**Table 2:** Comparison of clinical and radiographic factors in case and control groups. φ: phi coefficient; χ²: Pearson’s chi-square; Df: degrees of freedom; IQR: interquartile range; ^1^: Fisher’s exact test; ²: Mann–Whitney–Wilcoxon test

Factors	Case group	Control group	χ² statistic	Df	P-value	Effect size (φ)
Diabetes mellitus, n (%)	16 (40%)	11 (28%)	1.37	1	0.24	0.13
Arterial hypertension, n (%)	14 (35%)	16 (40%)	0.22	1	0.64	0.05
Dyslipidemia, n (%)	17 (43%)	13 (33%)	0.84	1	0.36	0.10
Thyroid dysfunction, n (%)	1 (3%)	3 (8%)	—	—	0.61^1^	—
High-risk occupation, n (%)	32 (80%)	17 (43%)	10.83	1	0.001	0.37
Dominant side involvement, n (%)	36 (90%)	26 (65%)	7.27	1	0.007	0.30
History of shoulder trauma, n (%)	16 (40%)	11 (28%)	1.37	1	0.24	0.13
Smoking, n (%)	9 (23%)	7 (18%)	0.30	1	0.58	0.06
Regular alcohol use, n (%)	3 (8%)	4 (10%)	—	—	0.99^1^	—
Body mass index, median (IQR)	28.2 (25.6–30.8)	25.8 (23.9–27.7)	—	—	0.003²	—
Critical shoulder angle, median (IQR)	38.3° (36.2–40.1°)	34.3° (30.6–38.2°)	—	—	0.005²	—
Acromial index, median (IQR)	0.72 (0.67–0.75)	0.66 (0.60–0.73)	—	—	0.003²	—

**Figure 4:**
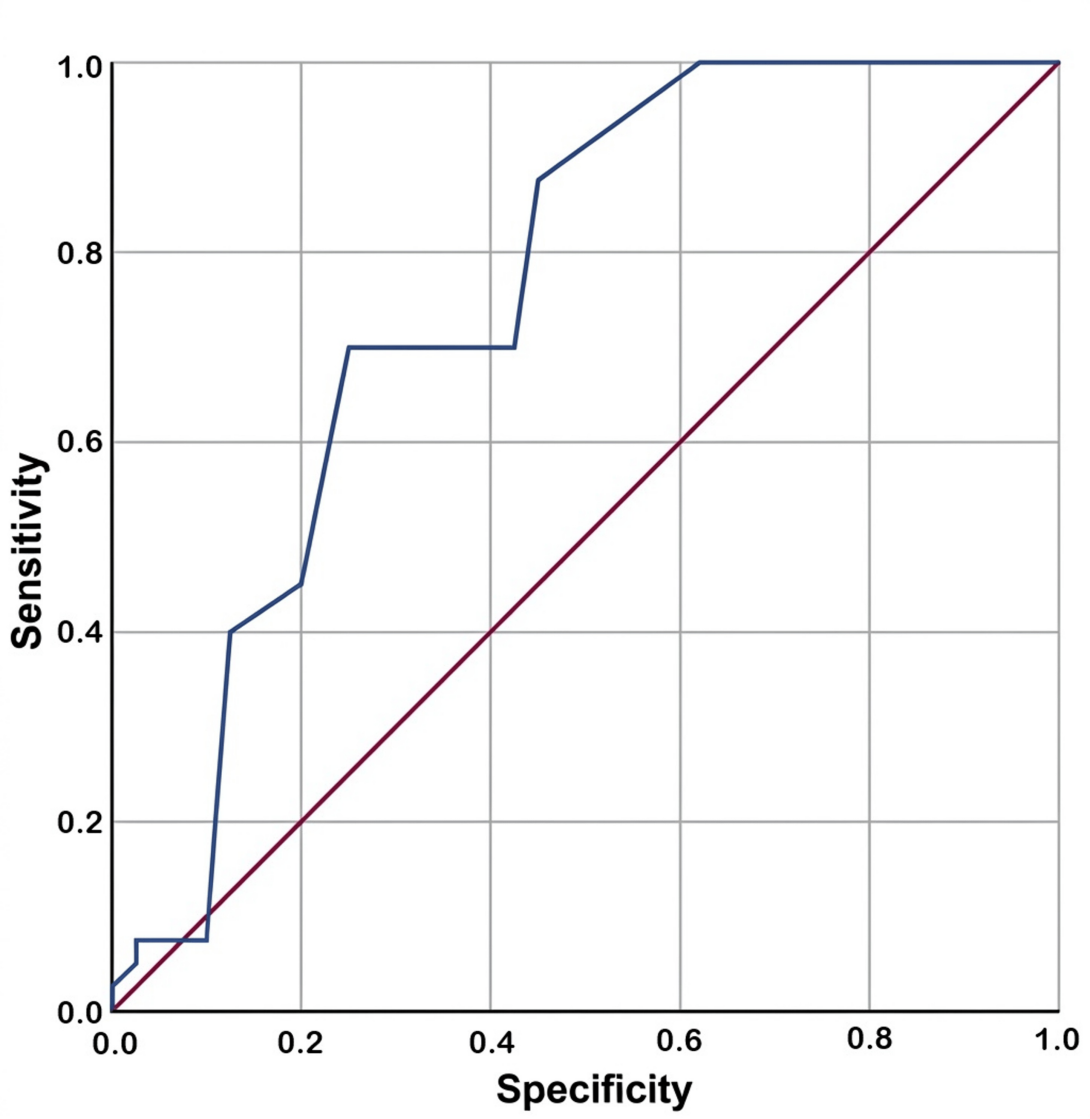
Receiver operating characteristic curve for critical shoulder angle.

**Figure 5:**
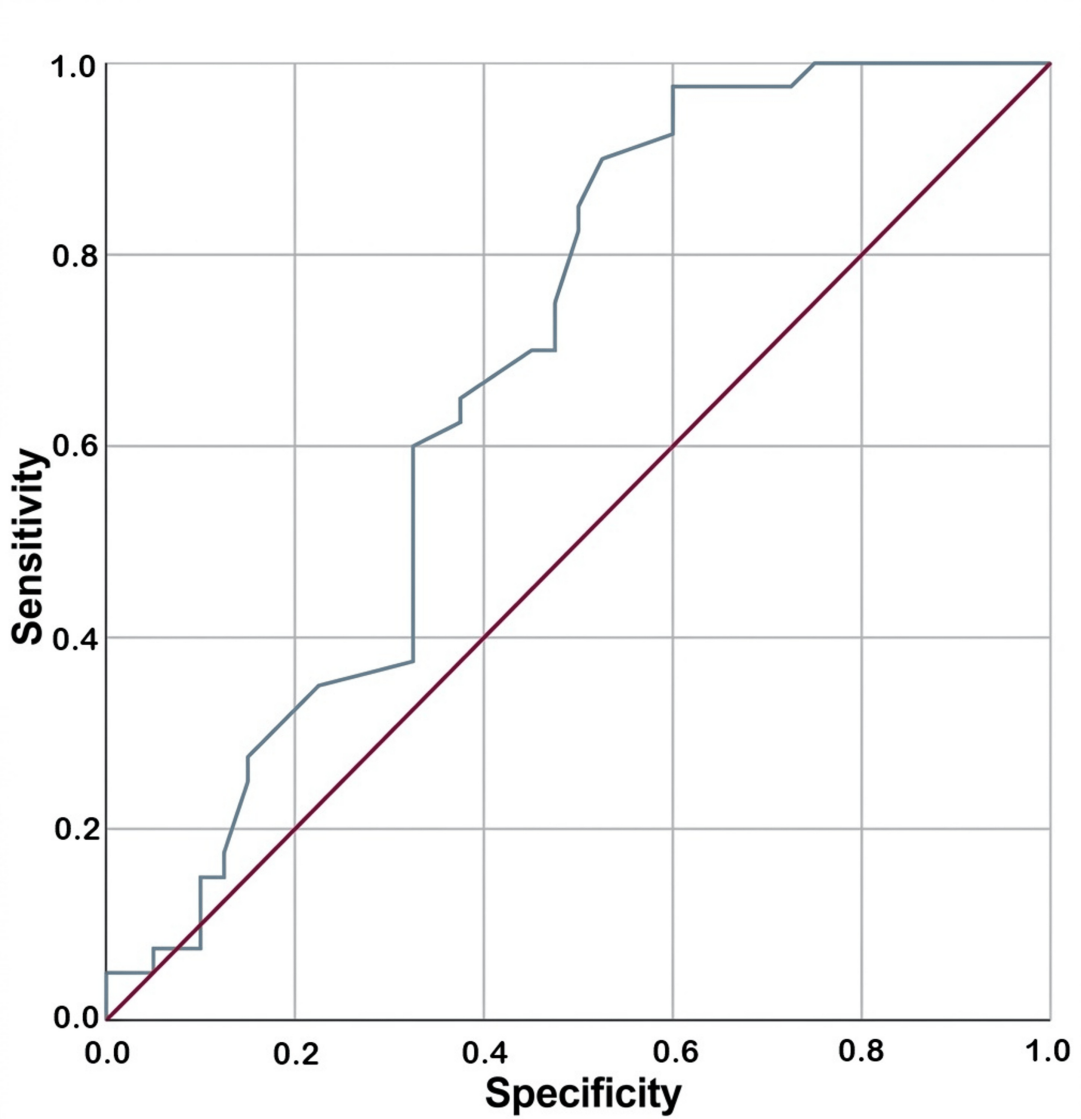
Receiver operating characteristic curve for acromial index.

ROC curve analysis demonstrated that the CSA (Figure 4) had superior predictive performance compared to the AI (Figure 5), yielding an area under the curve (AUC) of 0.755 (p < 0.001) versus 0.680 (p = 0.006), respectively.

A strong positive correlation was observed between the AI and CSA (r = 0.71, p < 0.01). Multivariable logistic regression analysis revealed that the CSA (odds ratio (OR) = 1.438; 95% confidence interval (CI) = 1.172-1.763; p = 0.001), BMI (OR = 1.266; 95% CI = 1.060-1.512; p = 0.01), dominant side involvement (OR = 4.458; 95% CI = 1.018-19.518; p = 0.047), and a high-risk occupation (OR = 8.379; 95% CI = 2.314-30.341; p = 0.001) were significant independent predictors of RCTs. The AI did not remain a significant independent predictor in the final model.

## Discussion

Demographics

The mean age of the participants in the case group was 56 years, which is lower than that reported in several published series [15,16]. Advanced age is a well-established risk factor for RCTs [12,17,18]. Women accounted for 65% of the cases, a proportion comparable to that reported by Lehman et al. [15], but higher than that observed by Yamamoto et al. [16].

Comorbidities

No significant association was observed between diabetes and RCT in our series, in contrast to most of the available literature [8,19]. This discrepancy may be explained by underdiagnosis of diabetes in our setting, highlighting the potential value of systematic screening.

Similarly, no significant relationship was identified between arterial hypertension and RCT. The literature remains conflicting: Sayampanathan and Andrew reported insufficient evidence to consider hypertension a risk factor [20], whereas Gumina et al. suggested hypertension-related peripheral hypoperfusion due to reflex vasoconstriction and described a correlation between hypertension severity and tear size [21].

Dyslipidemia was not significantly associated with RCT in our study, although several reports have shown higher total cholesterol, low-density lipoprotein, and triglyceride levels and lower high-density lipoprotein levels in affected patients [9,12,19,22]. The underlying pathophysiological mechanism remains uncertain.

Regarding thyroid dysfunction, no significant association was found. Although Oliva et al. suggested that tenocytes, through the presence of thyroid hormone receptors, may exhibit impaired reparative capacity in the setting of hormonal imbalance [10], most studies have concluded that there is no causal relationship [8,22].

Occupation

We identified a significant association between certain occupations and RCT, consistent with literature showing increased risk in agricultural and construction workers [22,23].

History of shoulder trauma

Distinguishing post-traumatic tears occurring on a degenerative rotator cuff from tears involving an otherwise healthy cuff remains challenging in routine clinical practice. The concept of acute-on-chronic RCTs has been described by Jeong et al. [8]. Mall et al. emphasized that traumatic tears on a previously healthy cuff tend to occur in younger patients [24].

Dominant side involvement

Dominant side involvement was significantly associated with the occurrence of RCT, in line with previous reports [16]. This relationship may be explained by the greater mechanical demand placed on the dominant upper limb, which may accelerate tendon overload and degenerative wear.

Smoking and alcohol consumption

No significant association was observed between smoking and RCT in our series, although most studies support a causal relationship [19,25]. Bishop et al. reported that smoking may accelerate tendon degeneration and is associated with larger tears [25]. Hatta et al. demonstrated that nicotine decreases matrix metalloproteinase-9 (MMP-9) and tissue inhibitor of metalloproteinases-3 expression as well as MMP-9 enzymatic activity, thereby disrupting the physiological remodeling of the tendon extracellular matrix [26].

We also found no significant association between alcohol consumption and RCT. Titchener et al. reported similar findings [27], whereas Passaretti et al. [7] observed higher alcohol intake among patients with tears, particularly massive tears, possibly through inhibition of fibroblast proliferation.

Body mass index

A higher BMI was significantly associated with RCT. Several authors have similarly reported an increased predisposition among obese patients [11,22,28]. Proposed mechanisms include tendon hypovascularity secondary to atherosclerosis and a systemic pro-inflammatory state driven by increased adipokine activity [28]. Reduced adiponectin levels and leptin resistance may further promote intracellular oxidative stress [11,22].

Acromial index

A higher AI was significantly associated with RCT. Nyffeler et al. [5] explained this relationship through a biomechanical mechanism: the middle deltoid, whose principal insertion is on the lateral acromion, generates an upward vertical force that is counterbalanced by the rotator cuff. Acromial morphology alters the “wrapping angle,” i.e., the extent to which the deltoid wraps around the greater tuberosity, thereby influencing the load transmitted to the cuff.

Critical shoulder angle

We demonstrated a significant association between a higher CSA and RCT. Moor et al. described the CSA as the integration of two predisposing factors: lateral acromial offset and superior glenoid inclination [6]. The CSA has the advantage of being simple to measure, reproducible, and independent of humeral positioning [14]. The median CSA of the case group in our series (38.3°) was comparable to values reported by Daggett et al. (37.9°), Spiegl et al. (37.3°), and Moor et al. (38°) [6,29,30]. The median CSA in the control group (34.3°) was close to values reported in healthy controls [6,29].

Strengths and limitations

This study has several strengths. It evaluates both clinical and radiographic factors within a matched case-control design, providing a comprehensive assessment of variables associated with RCTs. The use of multivariable logistic regression and ROC curve analysis strengthens the interpretation of the results, and age- and sex-matching helped limit confounding. In addition, the radiographic parameters were clearly defined and measured using a standardized approach.

However, several limitations should be acknowledged. The retrospective design does not allow causal inference and may introduce selection bias. The relatively small sample size may limit statistical power and result in wide CIs. Radiographic measurements were performed by a single observer, who was not blinded to the clinical status of the patients, which may represent a potential source of measurement bias. Inter- and intra-observer reliability were not assessed, and the use of manual measurements may introduce a degree of measurement error and observer-dependent variability compared to digital methods, although these measurements have been widely reported as reproducible in the literature [14]. Despite its high sensitivity in experienced hands, the use of ultrasound alone to exclude RCTs may have led to misclassification, particularly for partial-thickness tears. Finally, as a single-center study, the findings may not be fully generalizable to other populations.

## Conclusions

In this study, RCTs were significantly associated with BMI, dominant side involvement, high-risk occupational exposure, AI, and CSA. Among the anatomical parameters, the CSA demonstrated greater predictive value than the AI. These findings suggest that both anatomical and clinical factors may play a role in the occurrence of RCTs. However, due to the retrospective design, no causal relationships can be established. The results may have clinical implications for risk stratification and prevention, particularly regarding weight management and workplace ergonomics, although further prospective studies are needed to confirm these associations.
